# The seasonal changes of the gut microbiome of the population living in traditional lifestyles are represented by characteristic species-level and functional-level SNP enrichment patterns

**DOI:** 10.1186/s12864-021-07372-0

**Published:** 2021-01-28

**Authors:** Xue Zhu, Jiyue Qin, Chongyang Tan, Kang Ning

**Affiliations:** Key Laboratory of Molecular Biophysics of the Ministry of Education, Hubei Key Laboratory of Bioinformatics and Molecular-imaging, Department of Bioinformatics and Systems Biology, Center for AI Biology, College of Life Science and Technology, Huazhong University of Science and Technology, Wuhan, 430074 Hubei China

**Keywords:** Traditional lifestyle, Gut microbiome, SNP enrichment, Seasonal change

## Abstract

**Background:**

Most studies investigating human gut microbiome dynamics are conducted on humans living in an urban setting. However, few studies have researched the gut microbiome of the populations living traditional lifestyles. These understudied populations are arguably better subjects in answering human-gut microbiome evolution because of their lower exposure to antibiotics and higher dependence on natural resources. Hadza hunter-gatherers in Tanzania have exhibited high biodiversity and seasonal patterns in their gut microbiome composition at the family level, where some taxa disappear in one season and reappear later. Such seasonal changes have been profiled, but the nucleotide changes remain unexplored at the genome level. Thus, it is still elusive how microbial communities change with seasonal changes at the genome level.

**Results:**

In this study, we performed a strain-level single nucleotide polymorphism (SNP) analysis on 40 Hadza fecal metagenome samples spanning three seasons. With more SNP presented in the wet season, eight prevalent species have significant SNP enrichment with the increasing number of SNP calling by VarScan2, among which only three species have relatively high abundances. Eighty-three genes have the most SNP distributions between the wet season and dry season. Many of these genes are derived from *Ruminococcus obeum*, and mainly participated in metabolic pathways including carbon metabolism, pyruvate metabolism, and glycolysis.

**Conclusions:**

Eight prevalent species have significant SNP enrichments with the increasing number of SNP, among which only *Eubacterium biforme*, *Eubacterium hallii* and *Ruminococcus obeum* have relatively high species abundances. Many genes in the microbiomes also presented characteristic SNP distributions between the wet season and the dry season. This implies that the seasonal changes might indirectly impact the mutation patterns for specific species and functions for the gut microbiome of the population that lives in traditional lifestyles through changing the diet in wet and dry seasons, indicating the role of these variants in these species’ adaptation to the changing environment and diets.

**Supplementary Information:**

The online version contains supplementary material available at 10.1186/s12864-021-07372-0.

## Background

The advancement of next-generation sequencing and bioinformatics techniques has made accessible the genetic information of the entire microbial community. The human gut microbiome has gained increasing research interest because of its critical role in metabolism, host nutrition, immune function, and central nervous system [1, 2]. However, most studies investigating its dynamics have mainly focused on the industrialized populations, who are regularly exposed to antibiotics and whose subsistence depends on artificially produced crops and animal products [3–5]. In contrast, unindustrialized populations living traditional lifestyles, with lower exposure to antibiotics and higher dependence on natural resources, are arguably better subjects in answering ancient human-gut microbiome relationship [6]. Recent studies on Hadza hunter-gatherers have shed light on the gut microbiome’s dynamics and adaptive versatility to lifestyle changes [7, 8]. The Hadza gut microbial communities exhibit a high degree of biodiversity [8] and a pattern of seasonal cycling in microbiome composition, where some taxa (at the family level) disappear in one season and reappear at a later time [7]. The species in Hadza individuals are the most seasonally volatile and could differentiate industrialized and traditional populations [7, 9]. Although the SNP space associated with seasonal changes of the Hadza gut microbial communities have been profiled, the nucleotide changes remain unexplored at the genome level. It is unclear how microbial communities change at the genome level under environmental stressors caused by seasonal changes.

The genome-level variations of the gut microbial communities can be examined by SNP calling tools (e.g. GATK [10], BCFtools [11], VarScan2 [12]). As genomic variations such as substitution, translocation, deletion and insertion, can lead to changes in antibiotic resistance [13] or pathogenicity [14], which can indicate the response to selection pressures [15], it is interesting to perform a high-resolution investigation into the Hadza gut microbiome. Previously, genomic variants have been investigated by Schloissnig [16] on studies about microbiomes [17] and their association with human diseases [18] for the modern population. Here, we performed a strain-level SNP analysis of Hadza gut metagenome to decipher the microbiome dynamics from the perspective of SNP enrichment. We first evaluated the SNP calling methods on simulated metagenome datasets, and selected the best method, namely VarScan2, for SNP calling on Hadza gut metagenome. Then, we performed an in-depth analysis of the SNP enrichments in species and functions along with the seasonal shifts for the Hadza population, and attempted to interpret the enrichment profile and dynamic patterns for such enrichments.

## Results and discussions

### Assessment of SNPcalling tools

**Fig. 1:**
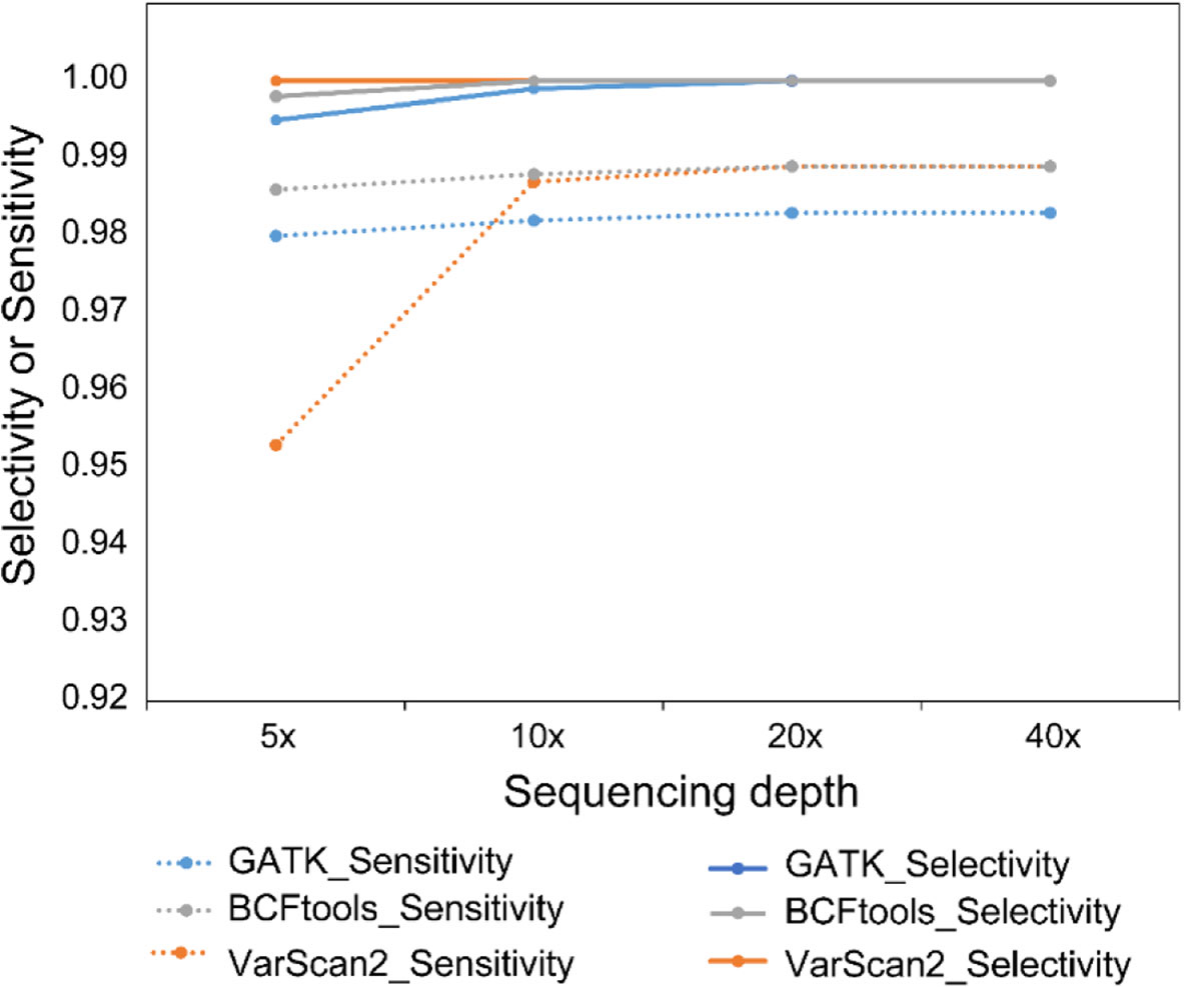
Comparison of selectivity and sensitivity of three SNP-calling tools (BCFtools, GATK and VarScan2) based on simulated reads with different sequencing depths. The x-axis represents the sequencing depth, and the y-axis displays the selectivity and the sensitivity of BCFtools, GATK and VarScan2

To select the most suitable variant-calling tool for the strain-level SNP analysis, we first evaluated three mainstream tools (GATK [10], BCFtools [11], VarScan2 [12]) based on their performance in terms of sensitivity and selectivity (Materials and Methods). A SNP list containing 10,786 sites from 5 major species residing in human gut (*Faecalibacterium prausnitzii* (reference genome size: 3,080,849 bp), *Prevotella copri* (3,507,873 bp), *Methanobrevibacter smithii* (1,853,160 bp), *Eubacterium biforme* (2,415,920 bp), *Treponema succinifaciens* (2,731,853 bp)) was used to generate a mutated reference genome set (Materials and Methods). Comparing the SNP identified by the three tools with the true SNP (Table 1 and Fig. 1), VarScan2 showed the highest selectivity (100%) at all sequencing depths, followed by BCFtools and GATK. In terms of sensitivity, at around 8x sequencing depth, VarScan2 showed the lowest sensitivity. Starting from the sequencing depth of 10x, GATK showed relatively lower sensitivity at all depths, while BCFtools and VarScan2 had almost the same sensitivity (approach 100%, except for 5x). Though we have acknowledged the limitations of this attempt to mimic human gut microbiome variation (e.g. the simplification by using only five species), we deem this evaluation as a useful guidance for the following Hadza gut microbiome analysis. Considering both selectivity and sensitivity, we chose VarScan2 for the Hadza gut metagenome SNP analysis.

**Table 1:** Comparison of the SNP identification results by the three tools. In these simulated data, the SNP coverages (defined as the number of SNPs identified by the software on these five genomes, divided by the sum of the five genome sizes) were also shown for different tools and different sequencing depths

Tool	Depth	Match	# Mismatch	# False positive	# False negative	# All SNPs	SNP Coverage (%)	Sensitivity (%)	Selectivity (%)
GATK	5x	10,567	0	52	218	10,619	0.078	98.0	99.5
VarScan2	5x	10,283	0	0	502	10,283	0.076	95.3	100.0
BCFtools	5x	10,635	0	24	150	10,659	0.078	98.6	99.8
GATK	10x	10,590	0	6	195	10,596	0.076	98.2	99.9
VarScan2	10x	10,647	0	0	138	10,647	0.078	98.7	100.0
BCFtools	10x	10,656	0	0	129	10,656	0.078	98.8	100.0
GATK	20x	10,599	0	0	186	10,599	0.078	98.3	1.000
VarScan2	20x	10,661	0	0	124	10,661	0.078	98.9	100.0
BCFtools	20x	10,663	0	0	122	10,663	0.078	98.9	100.0
GATK	40x	10,607	0	0	178	10,607	0.078	98.3	100.0
VarScan2	40x	10,667	0	0	118	10,667	0.078	98.9	100.0
BCFtools	40x	10,665	0	0	120	10,665	0.078	98.9	100.0

### Community compositions and seasonal changes of Hadza gut microbiome

**Fig. 2:**
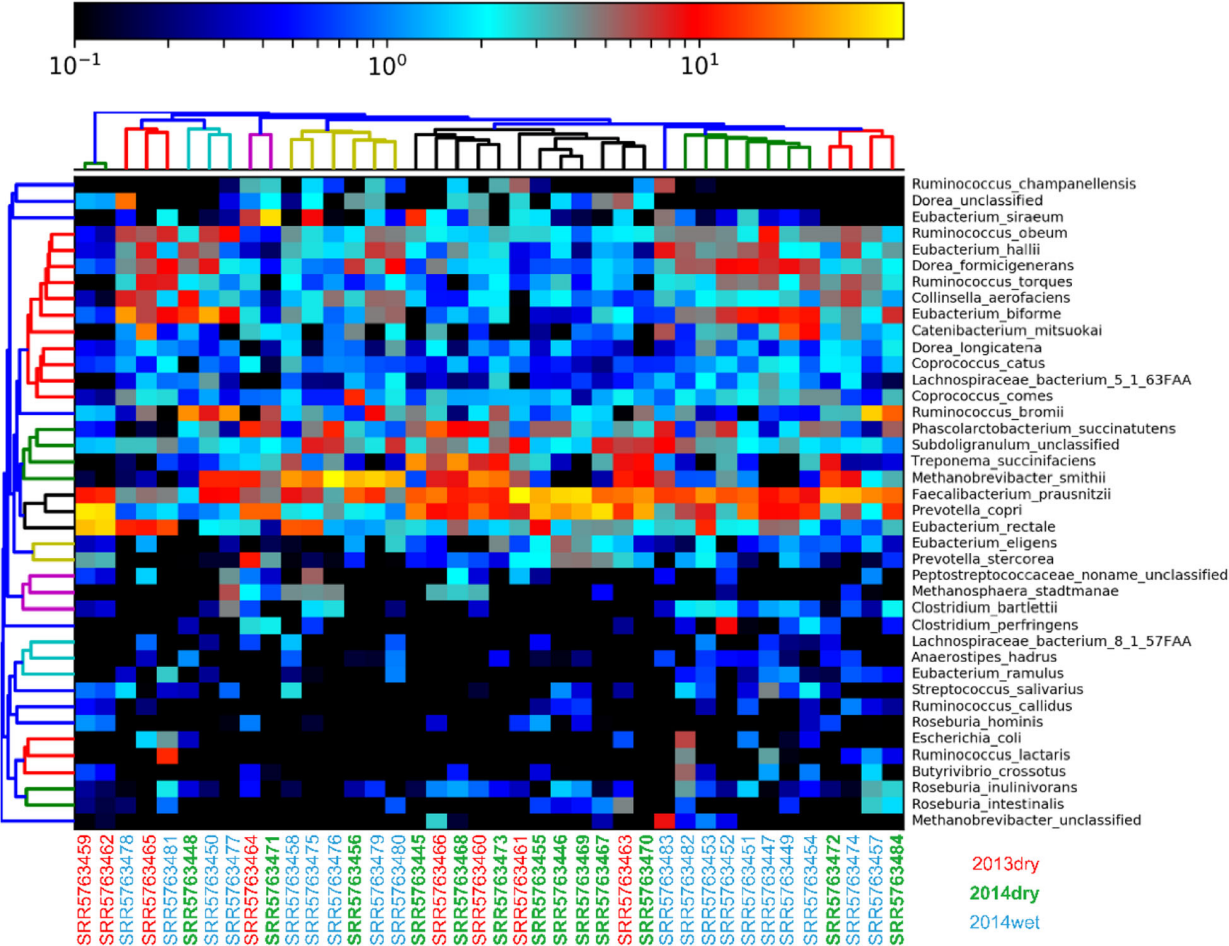
Heatmap of Hadza gut microbial community composition at the species level. Drawn by MetaPhlAn2 based on the species-abundance (log value) table, each row represents a species and each column represents a sample. Three colors were used to distinguish the samples from different seasons (red: samples collected in the dry season of 2013 (2013dry); green: samples collected in the dry season of 2014 (2014dry); blue: samples collected in the wet season of 2014 (2014wet)). Only the top 40 species with the highest abundance were shown here. The main species in Hadza gut include *Faecalibacterium prausnitzii*, *Prevotella copri*, *Methanobrevibacter smithii*, *Eubacterium biforme*, *Treponema succinifaciens*, which were represented in bright color (e.g., red, orange, yellow). The gradient color bar indicates, from left (dark blue) to right (yellow), the increasing abundance of these 40 species

A total of 116 species, including 16 unclassified species, were identified (Fig. 2), and the main species were *Faecalibacterium prausnitzii*, *Prevotella copri*, *Methanobrevibacter smithii*, *Eubacterium biforme*, *Treponema succinifaciens*. This result was in line with the previous report about the high abundance of *Prevotella* and *Treponema* [7, 8]. Although *Treponema* is famous for one species, namely *Treponema pallidum*, which could cause syphilis [19], here we found that *Treponema* in Hadza gut was dominated by *Treponema succinifaciens*, which plays a role in the hydrolysis of cellulose and xylose [20].

**Fig. 3:**
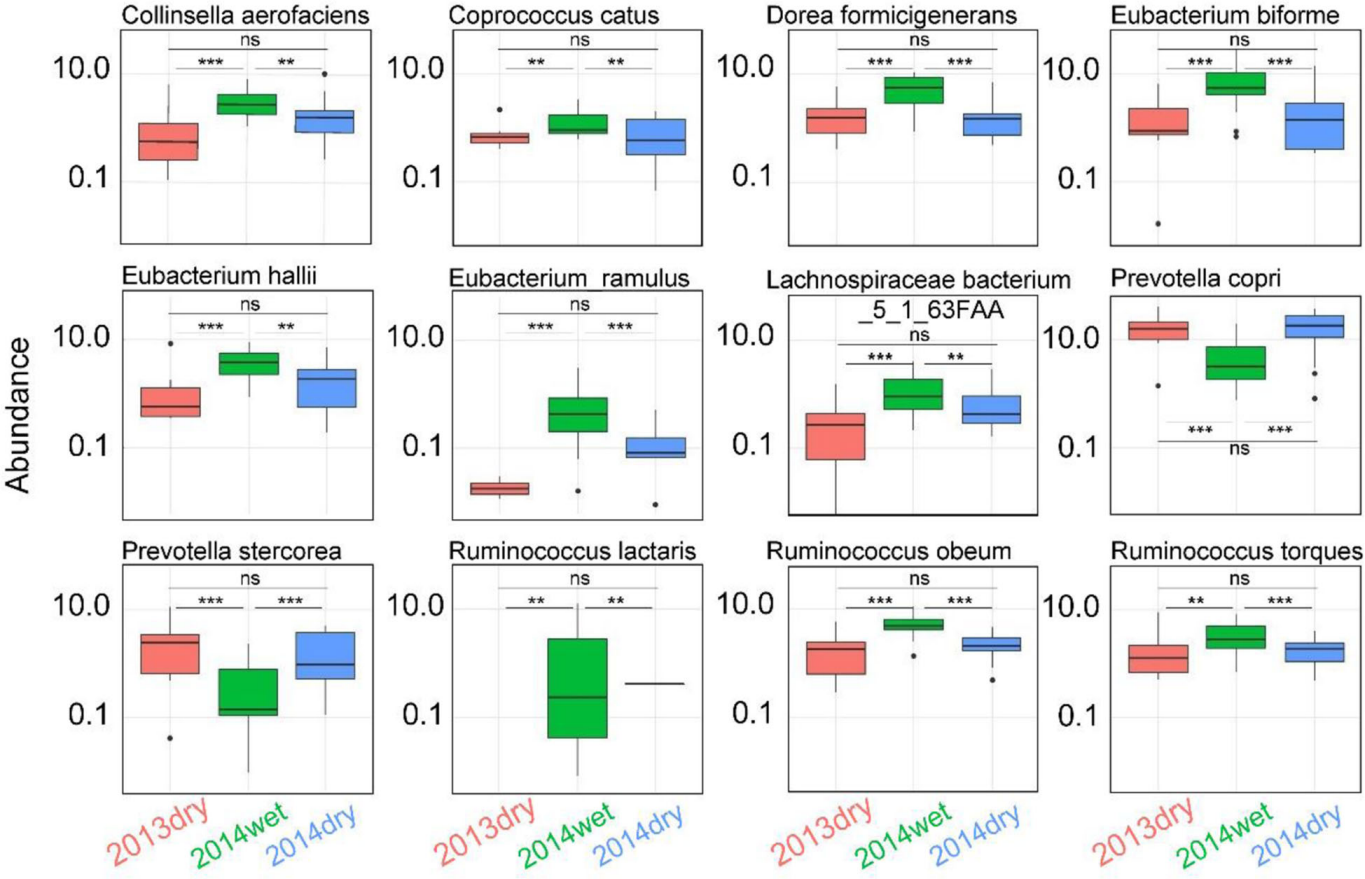
Abundance in certain gut microbial species differed among seasons. The changes in abundance across different seasons were shown with each panel representing one species. 2013dry samples (samples collected in the dry season of 2013; *n* = 8), 2014wet samples (samples collected in the wet season of 2014; *n* = 19), 2014dry samples (samples collected in the dry season of 2014; *n* = 13) were denoted by red, green and blue color, respectively. These 12 species have different (*P* < 0.05, Wilcoxon rank-sum test) abundance between wet and dry seasons, but they have indistinguishable abundance between adjacent dry seasons

From 116 identified species, we selected 33 species (Supplementary Table 1), which were present in at least 8 samples from at least one season, to analyze their abundance differences. Among which, the abundance of 12 species (Fig. 3, Supplementary Table 1) was significantly different (*P* < .05, Wilcoxon rank-sum test; Fig. 3) between the dry season of 2013 (2013dry) and the wet season of 2014 (2014wet) seasons, as well as between the dry season of 2014 (2014dry) and 2014wet seasons, but the abundance of 12 species was similar between 2013dry and 2014dry seasons. Among these 12 species, only the abundance of *Prevotella copri* and *Prevotella stercorea* decreased in 2014wet, which accords with the previous report of less *Prevotellaceae* in 2014wet [7]. *Prevotella copri* has been proved to act in glucose metabolism [21]. and its decreased abundance in the wet season may be associated with the Hadza population’s seasonal dietary changes. In contrast, the other 10 species showed higher abundance in 2014wet (Fig. 3), including *Ruminococcus obeum* (genome size: 2,607,950 bp) and *Ruminococcus lactaris* (2,729,735 bp), which belong to Firmicutes. Since a previous report has found that Firmicutes showed relatively stable abundance across seasonal succession [7]. The results suggested that in 2014wet, there might exist species belonging to Firmicutes that offset this difference.

### Strain-level SNP enrichment with the seasonal succession

**Fig. 4:**
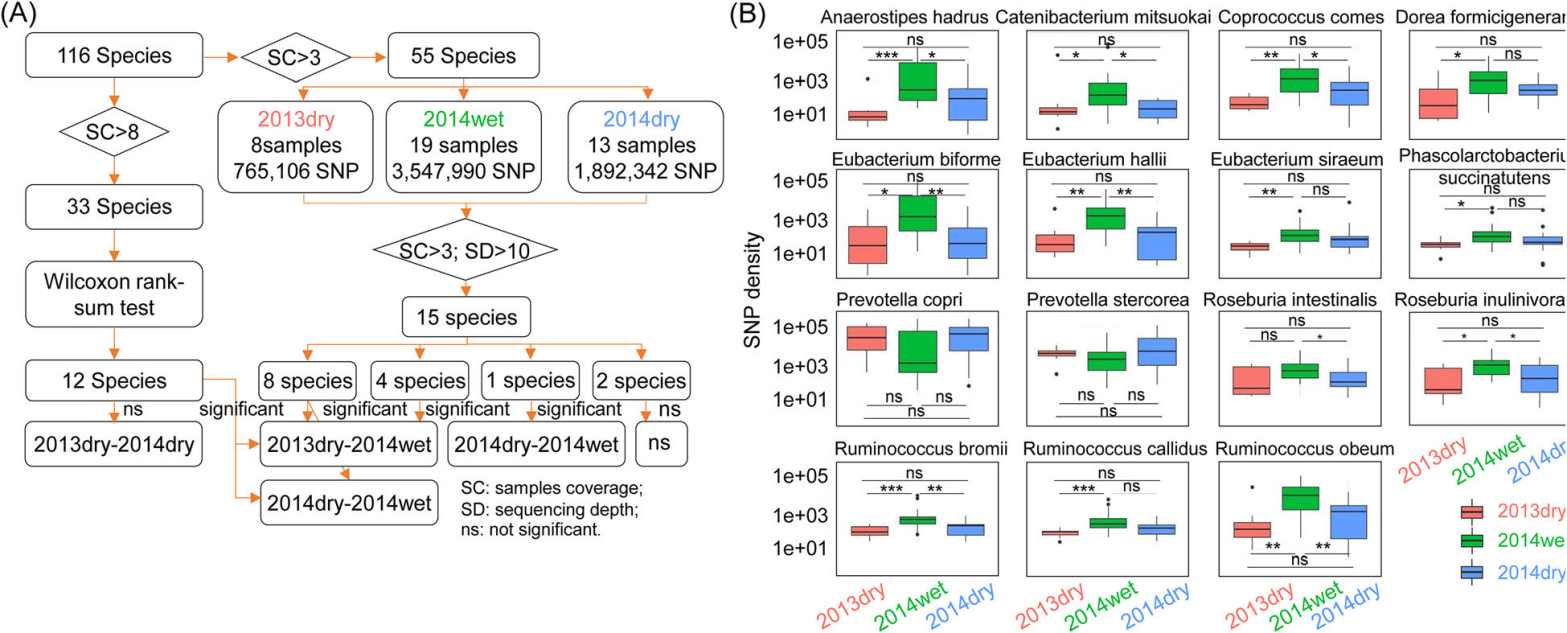
Certain species in the Hadza gut microbiome showed enrichment of SNPs in the wet season. **a** An overview of analysis workflow for both species enrichment (left) and SNP enrichment (right) in samples, and the SNP density comparison among the samples from three seasons. **b** The comparison of SNP density among the three seasons, with each panel representing one of the 15 prevalent species. Eight species showed more SNP in the wet season but comparable SNP in adjacent dry seasons. All boxes were tested by Wilcoxon rank-sum tests. *P < 0.05, **P < 0.01, ***P < 0.001; SC: sample coverage; SD: sequencing depth; n.s.: not significant

To explore SNP changes with the seasonal succession, we selected 55 most prevalent species from 116 species to be included in the reference genome set. After threshold filtering (>10x sequencing depth, and sequencing quality score > 15), we identified 765,106 SNP (8 samples, avg. 95,638 SNP) in samples collected in the dry season of 2013 (2013dry samples), 3,647,990 SNP (19 samples, avg. 191,999 SNP) in samples collected in the wet season of 2014 (2014wet samples), and 1,892,342 SNP (13 samples, avg. 145,564 SNP) in samples collected in the dry season of 2014 (2014dry samples) (Table 2). This result demonstrated that there were more SNP in the wet season, which motivated us to investigate where these genome variations mainly originated from 15 species, whose average sequencing depth was above 10x in at least 3 samples (Fig. 4 and Supplementary Table 2), were selected for further analysis. Computing their SNP density (namely SNP occurrence frequency) in each sample, we found that all species showed indistinguishable SNP density between dry seasons, while eight species (*Anaerostipes hadrus* (genome size: 3,172,613 bp), *Catenibacterium mitsuokai* (2,671,313 bp), *Coprococcus comes* (3,238,915 bp), *Eubacterium biforme*, *Eubacterium hallii* (2,722,180 bp), *Roseburia inulinivorans* (4,048,462 bp), *Ruminococcus bromii* (2,539,482 bp) and *Ruminococcus obeum*) showed SNP enrichment (*P* < .05, Wilcoxon rank-sum test) in the wet season compared to both dry seasons.

**Table 2:** Overview of the number of SNPs distributed in 2013dry, 2014wet and 2014dry

	# of SNP	# of Sample	Average
2013dry	765,106	8	95,638
2014wet	3,647,990	19	191,999
2014dry	1,892,342	13	145,565

The enrichment of SNPs in these species in the wet season was speculated to associate with the environmental and dietary changes in wet and dry seasons. The Hadza’s activities largely focus on food acquisition. In the wet season, berry foraging and honey consumption are more frequent, whereas hunting is more successful during the dry season [7]. For example, the high protein diet in the dry season can affect acetate fermentation, which can be the reason behind the reduced SNP density of *Ruminococcus bromii* in the dry season as *Ruminococcus bromii* is known as an acetate producer [22]. Moreover, five out of the eight species with more SNP in the wet season didn’t differ in abundance across seasons, which indicated that the increased genome variation we observed was not due to an increased abundance. In other words, SNP characteristics might be independent of species abundance, thus provided us with a new perspective for studying microbial community dynamics.

**Fig. 5:**
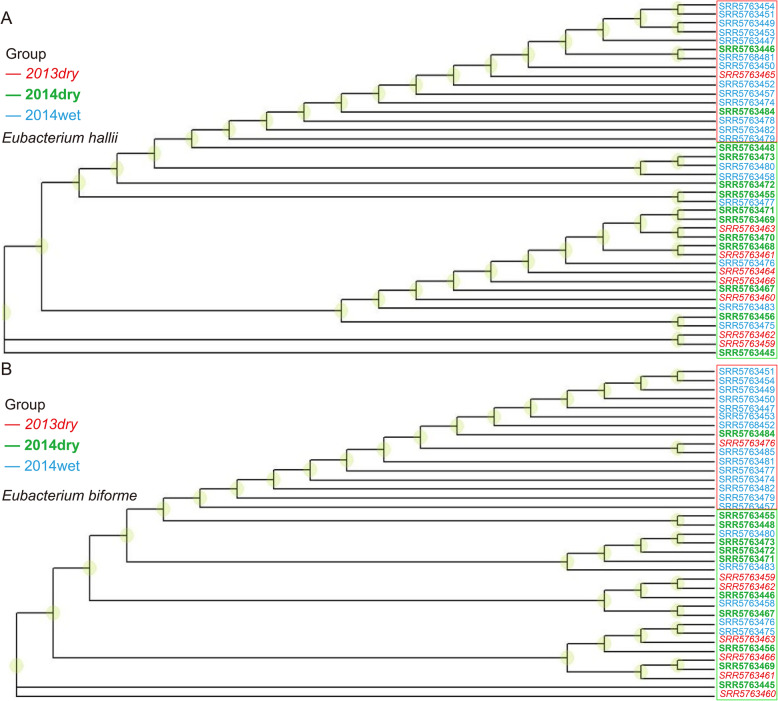
Wet-season samples were separable from dry-season samples in phylogenic trees. Phylogenic trees based on whole-genome SNP sites for two example species were shown here. Each branch represented a sample, and samples from different seasons were differentiated by three colors (red: samples collected in the dry season of 2013 (2013dry); green: samples collected in the dry season of 2014 (2014dry); blue: samples collected in the wet season of 2014 (2014wet)). Clear clustering could be observed in both trees where wet-season samples (red box) were restricted in one cluster and dry-season samples in the other cluster (green box). **a** *Eubacterium hallii* species. Thirteen wet-season samples (68.42% of total wet-season samples) were in one cluster (red box) and 18 dry-season samples (85.71% of total dry-season samples) were in the other cluster (green box). **b** *Eubacterium biforme* species. Fourteen wet-season samples (73.68%) were in one cluster (red box) and 16 dry-season samples (88.89%) were in the other cluster (green box)

To understand whether SNP characteristics change among seasons, for each of the eight identified species mentioned above, phylogenic trees were constructed by RAxML [23] based on whole genome SNP sites with mutated allele frequency bigger than 0.5 (see Materials and Methods) and visualized by R package “ggtree”. Two example trees for *Eubacterium hallii* and *Eubacterium biforme* were shown (Fig. 5). Clear clustering could be observed in Fig. 5, where most of the wet-season samples were restricted in one cluster, and the dry-season samples) in the other (Fig. 5). *Eubacterium hallii* was present in all the 40 samples (Fig. 5a). Among them, 13 wet-season samples (68.42% of total wet-season samples) were in one cluster (red box) and 18 dry-season samples (85.71% of total dry season samples) were in the other cluster (green box) (Fig. 5a). Similarly, *Eubacterium hallii* was present in 37 samples (Fig. 5a). Among them, 14 wet-season samples (73.68%) were in one cluster (red box) and 16 dry-season samples (88.89%) were in the other (green box) (Fig. 5b). The phylogenic trees for the remaining six species were shown in Supplementary Fig. 1 and a similar clustering could be observed. Interestingly, we found that one dry-season sample (SRA run: SRR5763465) was mixed with other wet-season samples for most of the species. Looking into its subject information revealed that this sample was from a 5-year-old child, who might have special SNP characteristics.

**Fig. 6:**
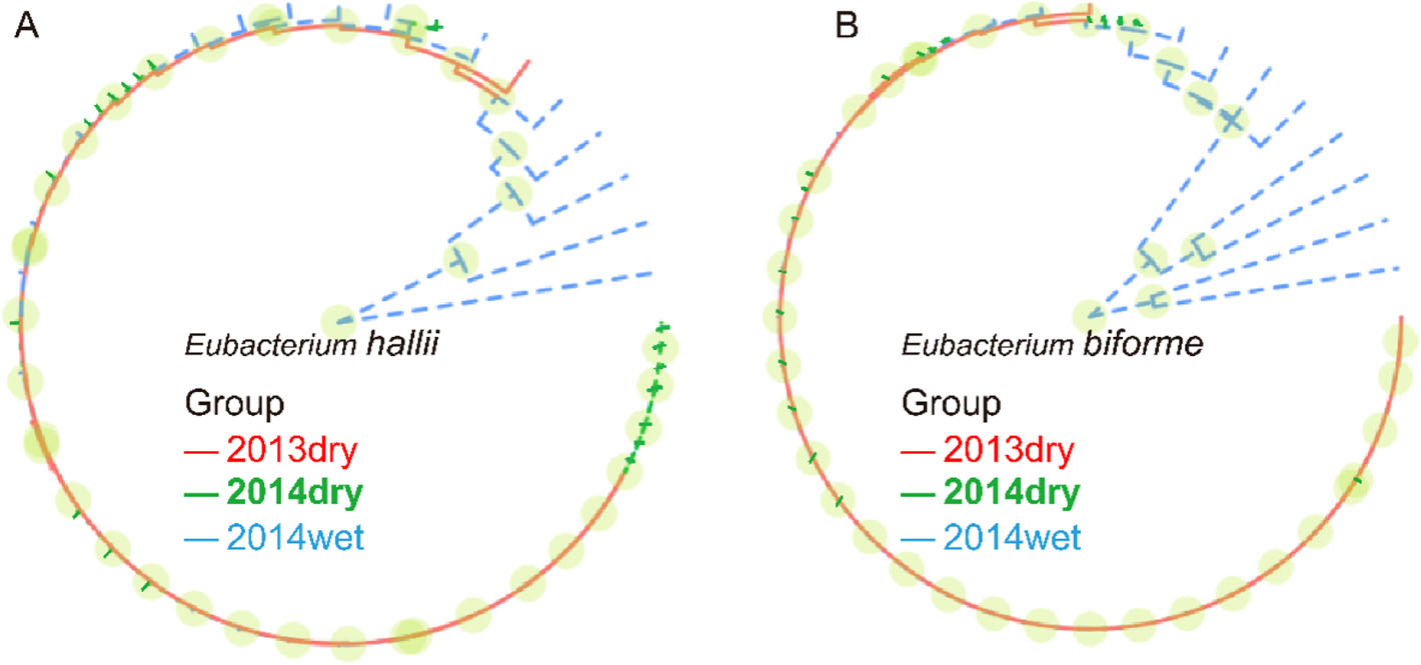
Wet-season samples were separable from dry-season samples based on their SNP profiles. **a** *Eubacterium hallii* species*.* **b** *Eubacterium biform* species*.* Clustering results based on whole-genome SNP sites for these two species were shown here. Each branch represented one sample, and samples from different seasons were differentiated by three colors (red: 2013dry; green: 2014dry; blue: 2014wet)

We also extracted the SNP sites with the mutated allele frequency > 0.5, and calculated the pairwise distance between samples using Manhattan distance. Hierarchical clustering was then used to cluster all the samples. The results for *Eubacterium biforme* and *Eubacterium hallii* were shown in Fig. 6 and the other six species (*Ruminococcus bromii*, *Ruminococcus obeum, Anaerostipes hadrus*, *Coprococcus comes*, *Catenibacterium mitsuokai*, *Roseburia inulinivorans*) were shown in Supplementary Fig. 2. Consistent with the above results in phylogenic trees, wet-season samples (blue) were separable from dry-season samples, while 2013dry samples (red) were mixed with 2014dry samples (green). The results also indicated the different SNP enrichment patterns in the wet season.

### Gene-level SNP enrichment with the succession of seasons

**Fig. 7:**
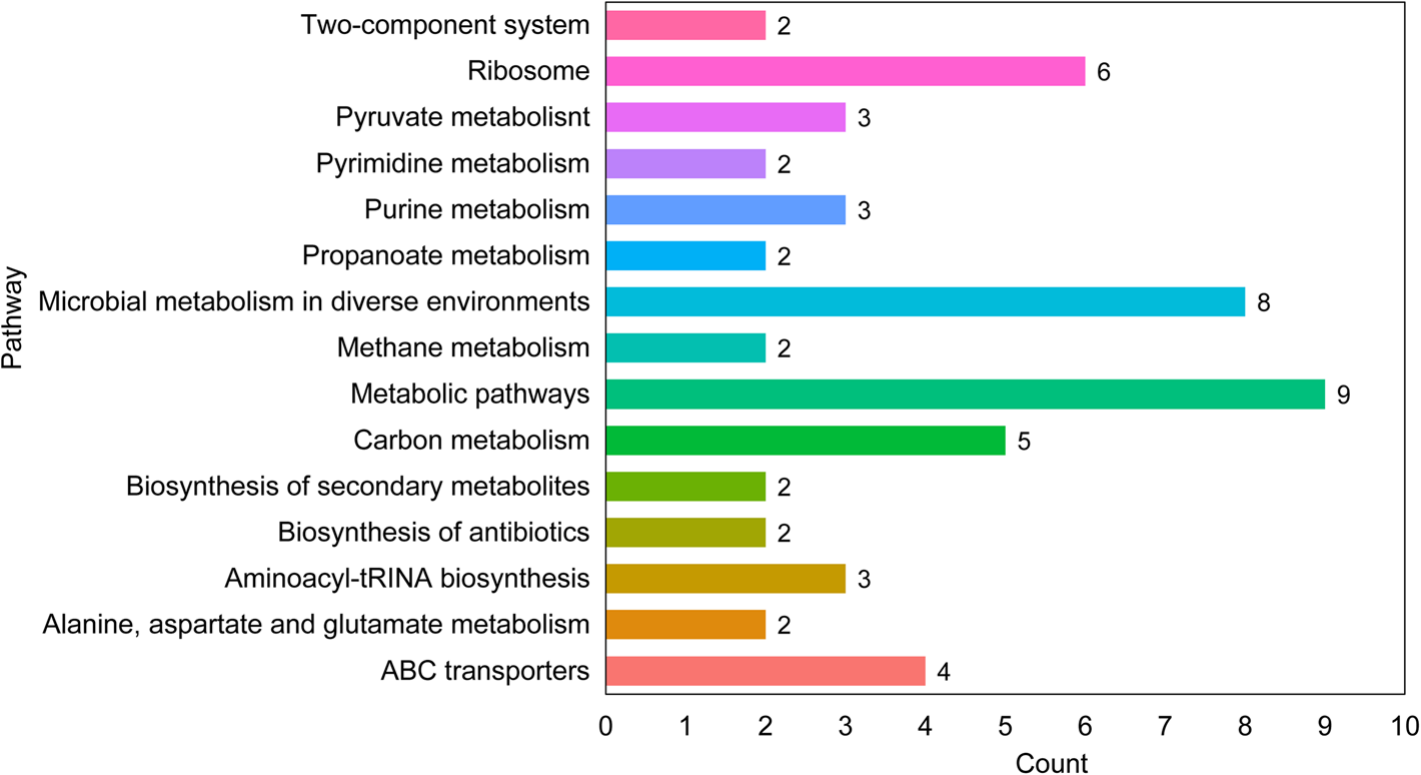
Pathway analysis of genes with cyclic SNP density patterns across seasons. Each bar represents a specific biological pathway and the number above it denotes the number of genes involved in this pathway. The genes have different SNP densities (*P* < 0.01, Wilcoxon rank-sum test) between the wet season and the dry season, but no difference between adjacent dry seasons, they mainly participate in metabolic pathways like carbon metabolism and ribosomes

From a total of 23,504 genes in the 15 selected species, 5959 genes whose SNP was present in at least 8 samples were targeted for further analysis. Eight hundred twenty-seven genes showed a significant difference in SNP density between the wet season and the dry season (*P* < 0.05, Wilcoxon rank-sum test), but no difference between adjacent dry seasons. Considering multiple comparison, we focused on the 83 genes, which achieved a significance at *P* < 0.01 level (Supplementary Table 3). For each of the 83 genes, the protein sequence was used to search by BLAST against the KEGG, and we selected the best hit as its pathway information. Thirty-six genes were annotated to 52 KEGG pathways (Supplementary Table 4), and the pathways that involve at least two genes were shown in Fig. 7. Many of these genes were from *Ruminococcus obeum*, concentrating on metabolic pathways, such as carbon metabolism, pyruvate metabolism and glycolysis (Fig. 7). This suggested that the seasonal changes might indirectly affect the mutation patterns for specific species, especially in core metabolic pathways, indicating the role of these variants in their adaptation to the changing environments and diets.

## Conclusions

This work has implications from both technical and microbiological viewpoints. Technically, we identified that VarScan2 showed comparatively better performance for SNP calling from the simulated metagenomic dataset, in both selectivity and sensitivity.. This analysis has provided a guidance for future investigation in variant calling and suggests that the direction to improve variant-calling tools is to improve the identification efficiency and reduce false-positive results at the same time.

From a microbiological viewpoint, we have identified SNPs’ characteristic enrichment in species and functions with the seasonal successions. Eight species (namely, *Anaerostipes hadrus, Catenibacterium mitsuokai, Coprococcus comes, Eubacterium biforme, Eubacterium hallii, Roseburia inulinivorans, Ruminococcus bromii* and *Ruminococcus obeum*) showed higher SNP density in the wet season (Fig. 4), in which only three (*Eubacterium biforme*, *Eubacterium hallii* and *Ruminococcus obeum*) (Fig. 3) had relatively high abundance. Phylogenic trees and hierarchical clustering of their whole genome SNP demonstrated the new strains emerged in the wet season. Additionally, we identified 83 genes with a highly significant difference in SNP density between the wet season and the dry season. Many of these genes were from *Ruminococcus obeum*, and were involved in metabolic pathways such as carbon metabolism, pyruvate metabolism and glycolysis. These results demonstrated a cyclic pattern in species abundance, SNP density at the species level, and SNP density at the genome level across three successive seasons, where the dry season was differentiated from the wet season, but the adjacent dry seasons were similar. This could be linked to the cyclic pattern in dietary and environmental factors across the seasons in this population living a traditional lifestyle. Highlighting the quantification and characterization of SNP, this work has also laid a foundation for future investigation of gut microbiome dynamics in response to changes in lifestyle and other environmental stressors.

## Methods

### The process for creating the simulated datasets containing the SNP list

For simulated data, we first generated a list containing 10,786 SNP sites from five major species (*Faecalibacterium prausnitzii, Prevotella copri, Methanobrevibacter smithii, Eubacterium biforme, Treponema succinifaciens*), which commonly reside in human gut and play specific functions [20, 24–27]. Then, a mutated reference genome set was achieved by replacing corresponding SNP sites. To create the SNP list for the five major species, we used BBMap randomreads to simulate the NGS data using muta_genome_combine.fna as “ref” value, used “length” parameter (set as 75) to specify the length of the reads, and used “reads” parameter (set as 1,000,000) to specify the number of generated reads, and set paired as true. The parameters “snprate”, “insrate”, and “delrate” specify the sequencing error model, representing the SNP frequency (set as 0.002), insertion frequency (set as 0.00002), and deletion frequency (set as 0.00002), “simplenames” specifies the reads name and set as t, and “addslash” parameter specifies (set as t), and it assigned using ‘/’ to distinguish the double-ended reads. The number of reads depends on the sequencing depth, with 1,000,000 reads for 5x, 2,000,000 reads for 10x, 4,000,000 reads for 20x, and 8,000,000 reads for 40x.

### Collection of real datasets

Hadza gut microbiome dataset includes whole-genome sequencing data that are described in [7]. The whole metagenomic sequencing data of Haza human gut microbiomes were downloaded from the NCBI SRA database with accession number SRA582120. Among 40 samples, eight are from the 2013dry season, 19 samples are from the 2014wet season, and 13 samples are from the 2014dry season. We first applied Trimmomatic [28] to remove the adapters and the low-quality bases of the raw dataset. MetaPhlAn2 [29] was utilized to determine the bacterial species and their abundance in each sample, then hclust2 was used to draw the heatmap of Hadza gut microbial community composition based on the merged abundance table.

### SNP calling by BCFtools, GATK and VarScan2

The processed bam files were used for SNP calling by three tools. The command for BCFtools was “samtools mpileup -ugf genome_for_sim.fna dupfree_sim_meta.bam| bcftools call -vmO v -V indels -o bcftools_meta.vcf”, where smtools -u specified uncompressed file, −g specified output as bcf format, −f specified reference sequence file, BCFtools -v specified only output variant sites, −O v specified output file as uncompressed vcf format and -V indels specified ignoring indel.

For GATK, SAMtools faidx and picard CreateSequenceDictionary were used to generate .fai file and .dict file, respectively, to index the reference file. The command is “java -jar GenomeAnalysisTK.jar -T HaplotypeCaller -R genome_for_sim.fna -I dupfree_sim_meta.bam -o GATK_meta.vcf”.

For VarScan2, samtools mpileup was first used to output mpileup file and the command is: “java -jar VarScan.v2.3.9.jar mpileup2SNP sim_meta.mpileup --min-coverage 1 --output-vcf 1 --variants --min-reads2 1 --min-avg-qual 1 > varscan_meta.vcf, where --min-coverage specified the minimum sequencing depth,--variants specified only output variants,--min-reads2 specified the minimum number of reads that support the SNP, −-min-avg-qual specified the minimum sequencing quality.

### Sensitivity and selectivity calculating

Comparing the SNP identified by the three tools with the actual SNP (ground truth), we assessed their performance from two aspects which were calculated as follows.

$$ {\displaystyle \begin{array}{c}\mathrm{sensitivity}=\mathrm{match}/\mathrm{all}\\ {}\mathrm{selectivity}=1-\left(\mathrm{false}\ \mathrm{positive}+\mathrm{mismatch}\right)/\mathrm{all}\end{array}} $$

Here, the ‘match’ represents the number of actual SNP identified by the tool, while the ‘false positive’ represents the number of SNP identified by the tool were non-existent. ‘mismatch’ represents the number of actual SNP identified as a wrong base by the tool. ‘all’ represents the number of all actual SNP.

### Variant calling procedure

**Fig. 8:**
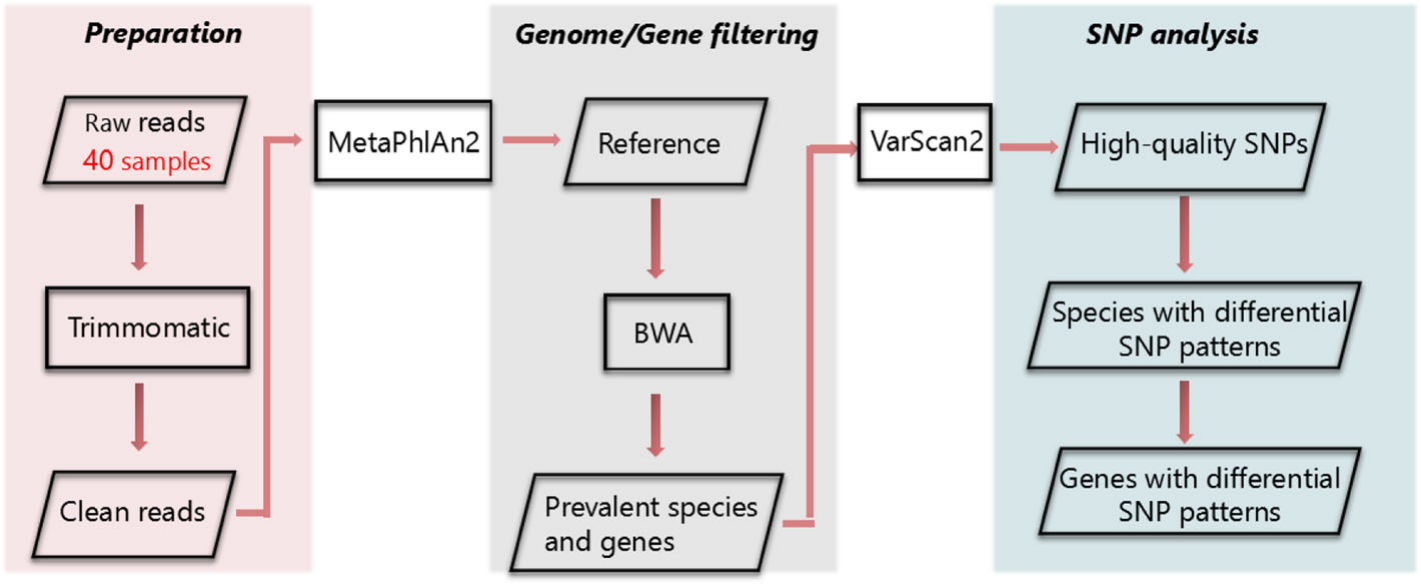
Variant calling procedure. Each colored block represented a series of analyses for data preparation, genome/gene filtration and SNP analysis

Clean reads of 40 samples were aligned to the reference genome set via BWA MEM with the parameter -R to specify the header group in output sam files. SAMtools view command was used with parameter -bS to transform sam files into bam files, and its sort command was used to sort bam files by the order of chromosomes. Picard MarkDuplicates command was used with parameter REMOVE_DUPLICATES = true to remove PCR duplicates. Samtools mpileup command was used with parameter -Bf to specify the reference genome set and output mpileup files. VarScan mpileup2SNP command was used with parameter --min-coverage 10 --output-vcf 1 --variants --min-avg-qual 15 to identify SNP from mpileup files and output vcf format files. The variant calling procedure was illustrated in Fig. 8.

### Assessment of SNP-calling tools

To select the most suitable variant-calling tool for the strain-level SNP analysis, we first evaluated three representative tools (GATK [10], BCFtools [11], VarScan2 [12]) based on their performance in terms of sensitivity and selectivity (see Materials and Methods). A SNP list containing 10,786 sites from 5 major species residing in human gut (*Faecalibacterium prausnitzii* (reference genome size: 3,080,849 bp), *Prevotella copri* (3,507,873 bp), *Methanobrevibacter smithii* (1,853,160 bp), *Eubacterium biforme* (2,415,920 bp), *Treponema succinifaciens* (2,731,853 bp)) was used to generate a mutated reference genome set. Then, BBMap was applied to generate paired-end simulated reads of length 75 with the sequencing error: SNPrate = 0.002, insrate = 0.00002, delrate = 0.00002. Different amounts of reads were generated to understand the effect of different sequencing depth, with 1,000,000 reads for 5x, 200,000 reads for 10x, 4,000,000 reads for 20x and 8,000,000 reads for 40x. We then used Burrows-Wheeler Aligner (BWA) to index the original reference genome set and MEM algorithm to align the simulated reads with the reference. The result files (in sam format) were converted to bam files by SAMtools [30], then sorted according to the header of the file and sequence in the file from left to right by the sort command. After that, we used Picard to remove PCR duplicates.

### Phylogenic tree construction and clustering

The parameters used for RAxML to construct phylogenic trees based on whole-genome SNP sites were -m ASC_GTRGAMMA -p 12345 --asc-corr = lewis -f a -× 12,345 -#100 and other parameters were set as default, where -m represents the specified nucleotide substitution model, −-asc-corr indicates the ascertainment bias correction method, and -# means the bootstrap numbers. The SNP sites whose mutated allele frequency is larger than 0.5 per sample were selected and calculated the pairwise distance between samples using Manhattan distance, and then clustered all the samples using hierarchical clustering. The clustering result was transformed into a tree file, then visualized in a circle tree.

### Wilcoxon rank-sum test

To detect whether the detected species or SNP between 2014dry and 2014wet, 2013dry and 2014dry, 2013dry and 2014wet, respectively, Wilcoxon rank-sum test was applied using R default package, wilcox.test function.

## Supplementary Information

**Additional file 1: Supplementary Table 1.** The Wilcoxon rank sum test results for the abundance of 33 main species in Hadza gut across three seasons. **Supplementary Table 2.** The Wilcoxon rank sum test results for the SNP density of 15 species in Hadza gut across three seasons. These 15 species are selected for enough sequencing depth in sufficent samples. Species with different (*P*<0.05) SNP density between wet and dry seasons but indistinct (*P*>0.05) between adjacent dry seasons are shown in boldface. **Supplementary Table 3.** Information of genes with characteristic SNP distribution pattern (*P*<0.01, Wilcoxon). **Supplementary Table 4.** Pathway information of 36 genes annotated to KEGG database. **Supplementary Figure 1.** Phylogenic trees based on whole genome SNP sites of remaining 6 species (mutated allele frequency exceeds 0.5). **Supplementary Figure 2.** Clustering results of remaining six species based on whole genome SNP sites (mutated allele frequency bigger than 0.2).

## Data Availability

The whole metagenomic sequencing data of Haza human gut microbiomes was downloaded from the NCBI SRA database with the accession number SRA582120.
