# Supplementary material for: The seasonal changes of the gut microbiome of the population living in traditional lifestyles are represented by characteristic species-level and functional-level SNP enrichment patterns
Source: BMC Genomics. 2021 Jan 28;22:83. doi: 10.1186/s12864-021-07372-0 (PMC7842071; doi:10.1186/s12864-021-07372-0)
Supplement: Supplementary file 1 — Additional file 1: Supplementary Table 1. The Wilcoxon rank sum test results for the abundance of 33 main species in Hadza gut across three seasons. Supplementary Table 2. The Wilcoxon rank sum test results for the SNP density of 15 species in Hadza gut across three seasons. These 15 species are selected for enough sequencing depth in sufficent samples. Species with different (P<0.05) SNP density between wet and dry seasons but indistinct (P>0.05) between adjacent dry seasons are shown in boldface. Supplementary Table 3. Information of genes with characteristic SNP distribution pattern (P<0.01, Wilcoxon). Supplementary Table 4. Pathway information of 36 genes annotated to KEGG database. Supplementary Figure 1. Phylogenic trees based on whole genome SNP sites of remaining 6 species (mutated allele frequency exceeds 0.5). Supplementary Figure 2. Clustering results of remaining six species based on whole genome SNP sites (mutated allele frequency bigger than 0.2). [file 12864_2021_7372_MOESM1_ESM.docx]

**Supplementary Materials**

**The seasonal changes of the gut microbiome of the population living in traditional lifestyles are represented by characteristic species-level and functional-level SNP enrichment patterns**

Xue Zhu^1,a^, Jiyue Qin^1,a^, Chongyang Tan^1^, Kang Ning^1,*^

*^1^ Key Laboratory of Molecular Biophysics of the Ministry of Education, Hubei Key Laboratory of Bioinformatics and Molecular-imaging, Department of Bioinformatics and Systems Biology, Center for AI Biology, College of Life Science and Technology, Huazhong University of Science and Technology, Wuhan, Hubei 430074, China*

These authors contributed equally to this work

^*^ Corresponding author. E-mail: ningkang@hust.edu.cn

**Supplementary Table 1. The Wilcoxon rank sum test results for the abundance of 33 main species in Hadza gut across three seasons.**

| **P_value** | **14dry_14wet** | **13dry_14dry** | **13dry_14wet** |
| --- | --- | --- | --- |
| *Anaerostipes_hadrus* | 0.2876 | 0.2741 | 0.03007 |
| *Butyrivibrio_crossotus* | 0.6978 | 0.4951 | 0.8841 |
| *Catenibacterium_mitsuokai* | 0.0983 | 0.6339 | 0.6137 |
| *Clostridium_bartlettii* | 0.0011 | 0.0897 | 0.0590 |
| *Collinsella_aerofaciens* | 0.0302 | 0.1403 | 0.0029 |
| *Coprococcus_catus* | 0.0272 | 0.6970 | 0.0133 |
| *Coprococcus_comes* | 0.9698 | 0.9155 | 0.5491 |
| *Dorea_formicigenerans* | 0.0002 | 1.0000 | 0.0065 |
| *Dorea_longicatena* | 0.3234 | 0.7501 | 0.7749 |
| *Eubacterium_biforme* | 0.0017 | 1.0000 | 0.0019 |
| *Eubacterium_eligens* | 0.0654 | 0.2315 | 0.8109 |
| *Eubacterium_hallii* | 0.0111 | 0.4137 | 0.0024 |
| *Eubacterium_ramulus* | 0.0015 | 0.7092 | 0.0022 |
| *Eubacterium_rectale* | 0.1950 | 0.1403 | 0.4184 |
| *Eubacterium_siraeum* | 0.2493 | 0.6374 | 0.6137 |
| *Faecalibacterium_prausnitzii* | 0.0369 | 0.5466 | 0.4815 |
| *Lachnospiraceae_bacterium_5_1_63FAA* | 0.0493 | 0.1687 | 0.0044 |
| *Methanobrevibacter_smithii* | 0.5142 | 0.8563 | 0.7749 |
| *Phascolarctobacterium_succinatutens* | 0.0197 | 0.5466 | 0.4184 |
| *Prevotella_copri* | 0.0022 | 1.0000 | 0.0065 |
| *Prevotella_stercorea* | 0.0024 | 0.7501 | 0.0086 |
| *Roseburia_hominis* | 0.1964 | 0.2235 | 0.0167 |
| *Roseburia_intestinalis* | 0.7525 | 0.7302 | 0.7302 |
| *Roseburia_inulinivorans* | 0.8779 | 0.8561 | 0.5066 |
| *Rothia_mucilaginosa* | 0.2214 | 0.6346 | 0.1289 |
| *Ruminococcus_bromii* | 0.2872 | 0.1403 | 0.7353 |
| *Ruminococcus_callidus* | 0.9668 | 0.9066 | 0.7968 |
| *Ruminococcus_champanellensis* | 0.5886 | 0.5886 | 0.9524 |
| *Ruminococcus_lactaris* | 0.0127 | 0.4925 | 0.0155 |
| *Ruminococcus_obeum* | 0.0001 | 0.3738 | 0.0015 |
| *Ruminococcus_torques* | 0.0081 | 0.8554 | 0.0359 |
| *Streptococcus_salivarius* | 0.2605 | 0.8260 | 0.4989 |
| *Treponema_succinifaciens* | 0.0407 | 0.3363 | 0.4257 |

**Supplementary Table 2. The Wilcoxon rank sum test results for the SNP density of 15 species in Hadza gut across three seasons.** These 15 species are selected for enough sequencing depth in sufficent samples. Species with different (P<0.05) SNP density between wet and dry seasons but indistinct (P>0.05) between adjacent dry seasons are shown in boldface.

| **P_value** | **13dry_14wet** | **13dry_14dry** | **14wet_14dry** |
| --- | --- | --- | --- |
| *Anaerostipes_hadrus* | 0.0007454 | 0.218 | 0.04594 |
| *Catenibacterium_mitsuokai* | 0.02945 | 0.8404 | 0.01502 |
| *Coprococcus_comes* | 0.00159 | 0.2685 | 0.03018 |
| *Dorea_formicigenerans* | 0.02802 | 0.2614 | 0.1258 |
| *Eubacterium_biforme* | 0.02516 | 0.8882 | 0.002703 |
| *Eubacterium_hallii* | 0.002932 | 0.7996 | 0.003992 |
| *Eubacterium_siraeum* | 0.006237 | 0.05489 | 0.2657 |
| *Phascolarctobacterium_succinatutens* | 0.03146 | 0.2935 | 0.1447 |
| *Prevotella_copri* | 0.1285 | 0.9718 | 0.1148 |
| *Prevotella_stercorea* | 0.2608 | 0.8596 | 0.2416 |
| *Roseburia_intestinalis* | 0.05832 | 0.4558 | 0.04075 |
| *Roseburia_inulinivorans* | 0.01125 | 0.4688 | 0.03693 |
| *Ruminococcus_bromii* | 0.0004171 | 0.3847 | 0.003451 |
| *Ruminococcus_callidus* | 0.0004171 | 0.2181 | 0.05264 |
| *Ruminococcus_obeum* | 0.00349 | 0.4688 | 0.004546 |

**Supplementary Table 3. Information of genes with characteristic SNP distribution pattern (P<0.01, Wilcoxon).**

| **species** | **id** | **product** |
| --- | --- | --- |
| *Roseburia_intestinalis* | WP_006858252.1 | sensor histidine kinase |
| *Roseburia_intestinalis* | WP_006858254.1 | ABC transporter permease |
| *Roseburia_intestinalis* | WP_006858468.1 | fibronectin |
| *Eubacterium_hallii* | WP_005345320.1 | hypothetical protein |
| *Eubacterium_hallii* | WP_044922469.1 | alpha/beta hydrolase |
| *Eubacterium_hallii* | WP_005347213.1 | sugar O-acetyltransferase |
| *Eubacterium_hallii* | WP_005348521.1 | sensor histidine kinase |
| *Prevotella_stercorea* | WP_007901928.1 | hydrophobe/amphiphile efflux-1 family RND transporter |
| *Coprococcus_comes* | WP_008373714.1 | hypothetical protein |
| *Dorea_formicigenerans* | WP_040015255.1 | hypothetical protein |
| *Dorea_formicigenerans* | WP_040015389.1 | MerR family DNA-binding transcriptional regulator |
| *Dorea_formicigenerans* | WP_005332103.1 | transposase |
| *Dorea_formicigenerans* | WP_040015578.1 | transposase |
| *Dorea_formicigenerans* | WP_040015583.1 | helix-turn-helix domain-containing protein |
| *Dorea_formicigenerans* | WP_005333755.1 | TnpV protein |
| *Dorea_formicigenerans* | WP_005334798.1 | DUF4316 domain-containing protein |
| *Phascolarctobacterium_succinatutens* | WP_040564899.1 | plasmid recombination protein |
| *Ruminococcus_obeum* | WP_044924544.1 | lysine--tRNA ligase |
| *Ruminococcus_obeum* | WP_005421747.1 | formate C-acetyltransferase |
| *Ruminococcus_obeum* | WP_005421991.1 | IS200/IS605 family transposase |
| *Ruminococcus_obeum* | WP_005422114.1 | ABC transporter ATP-binding protein |
| *Ruminococcus_obeum* | WP_005423122.1 | MATE family efflux transporter |
| *Ruminococcus_obeum* | WP_005423288.1 | DNA-directed RNA polymerase subunit beta |
| *Ruminococcus_obeum* | WP_005423313.1 | elongation factor Tu |
| *Ruminococcus_obeum* | WP_044925152.1 | hybrid sensor histidine kinase/response regulator |
| *Ruminococcus_obeum* | WP_005423870.1 | chromosomal replication initiator protein DnaA |
| *Ruminococcus_obeum* | WP_005424267.1 | xylulokinase |
| *Ruminococcus_obeum* | WP_005424767.1 | molecular chaperone DnaK |
| *Ruminococcus_obeum* | WP_044925681.1 | valine--tRNA ligase |
| *Ruminococcus_obeum* | WP_005424973.1 | 50S ribosomal protein L3 |
| *Ruminococcus_obeum* | WP_005424974.1 | 50S ribosomal protein L4 |
| *Ruminococcus_obeum* | WP_005425364.1 | formate--tetrahydrofolate ligase |
| *Ruminococcus_obeum* | WP_044925744.1 | CO dehydrogenase/CO-methylating acetyl-CoA synthase complex subunit beta |
| *Ruminococcus_obeum* | WP_005425650.1 | methionine--tRNA ligase |
| *Ruminococcus_obeum* | WP_005425942.1 | UDP-glucose--hexose-1-phosphate uridylyltransferase |
| *Ruminococcus_obeum* | WP_005426102.1 | glyoxalase |
| *Ruminococcus_obeum* | WP_082246510.1 | hypothetical protein |
| *Ruminococcus_obeum* | WP_044925873.1 | isoleucine--tRNA ligase |
| *Ruminococcus_obeum* | WP_005426440.1 | elongation factor Ts |
| *Ruminococcus_obeum* | WP_005426518.1 | rlx protein |
| *Ruminococcus_obeum* | WP_005426537.1 | DUF3849 domain-containing protein |
| *Ruminococcus_obeum* | WP_005426555.1 | peptidase |
| *Ruminococcus_obeum* | WP_044925952.1 | phosphoenolpyruvate carboxykinase (ATP) |
| *Ruminococcus_obeum* | WP_005427644.1 | ABC transporter permease |
| *Ruminococcus_obeum* | WP_005427646.1 | sugar ABC transporter ATP-binding protein |
| *Ruminococcus_obeum* | WP_022387934.1 | ABC transporter permease |
| *Ruminococcus_obeum* | WP_005427731.1 | class II aldolase family protein |
| *Ruminococcus_obeum* | WP_005427881.1 | 50S ribosomal protein L20 |
| *Ruminococcus_obeum* | WP_044926226.1 | transposase |
| *Ruminococcus_obeum* | WP_044926262.1 | hypothetical protein |
| *Ruminococcus_obeum* | WP_022388057.1 | electron transport complex subunit RsxC |
| *Ruminococcus_obeum* | WP_005428219.1 | cell surface protein |
| *Ruminococcus_obeum* | WP_005428668.1 | proline--tRNA ligase |
| *Ruminococcus_obeum* | WP_005421511.1 | DNA polymerase III subunit alpha |
| *Ruminococcus_obeum* | WP_004841179.1 | hypothetical protein |
| *Ruminococcus_obeum* | WP_005422095.1 | hypothetical protein |
| *Ruminococcus_obeum* | WP_005422239.1 | 1%2C3-beta-galactosyl-N-acetylhexosamine phosphorylase |
| *Ruminococcus_obeum* | WP_005423866.1 | 50S ribosomal protein L34 |
| *Ruminococcus_obeum* | WP_005423911.1 | sensor histidine kinase |
| *Ruminococcus_obeum* | WP_005424878.1 | peptide ABC transporter substrate-binding protein |
| *Ruminococcus_obeum* | WP_005425256.1 | 50S ribosomal protein L11 |
| *Ruminococcus_obeum* | WP_005425370.1 | carbon monoxide dehydrogenase |
| *Ruminococcus_obeum* | WP_005425377.1 | carbon-monoxide dehydrogenase catalytic subunit |
| *Ruminococcus_obeum* | WP_022389420.1 | phosphate acetyltransferase |
| *Ruminococcus_obeum* | WP_044925805.1 | glutamine-hydrolyzing GMP synthase |
| *Ruminococcus_obeum* | WP_005426079.1 | lactoylglutathione lyase |
| *Ruminococcus_obeum* | WP_005426100.1 | peptide deformylase |
| *Ruminococcus_obeum* | WP_005426368.1 | glutamine synthetase |
| *Ruminococcus_obeum* | WP_044925893.1 | glutamate synthase large subunit |
| *Ruminococcus_obeum* | WP_005426544.1 | hypothetical protein |
| *Ruminococcus_obeum* | WP_005427643.1 | LacI family transcriptional regulator |
| *Ruminococcus_obeum* | WP_005428565.1 | 30S ribosomal protein S11 |
| *Ruminococcus_obeum* | WP_005428696.1 | adenylosuccinate lyase |
| *Eubacterium_siraeum* | WP_005354788.1 | type I glyceraldehyde-3-phosphate dehydrogenase |
| *Anaerostipes_hadrus* | WP_008705329.1 | hypothetical protein |
| *Roseburia_inulinivorans* | WP_007884226.1 | IS200/IS605 family transposase |
| *Roseburia_inulinivorans* | WP_007884793.1 | hypothetical protein |
| *Roseburia_inulinivorans* | WP_015542402.1 | hypothetical protein |
| *Eubacterium_biforme* | WP_003866316.1 | IS110 family transposase |
| *Eubacterium_biforme* | WP_003865806.1 | hypothetical protein |
| *Eubacterium_biforme* | WP_040466579.1 | hypothetical protein |
| *Eubacterium_biforme* | WP_083783565.1 | hypothetical protein |
| *Ruminococcus_callidus* | WP_021683572.1 | DNA-binding response regulator |

**Supplementary Table 4. Pathway information of 36 genes annotated to KEGG database.**

| **species** | **id** | **entry** | **pathway** |
| --- | --- | --- | --- |
| *Ruminococcus_obeum* | WP_044924544.1 | EUR_28990 | Aminoacyl-tRNA biosynthesis |
| *Ruminococcus_obeum* | WP_005421747.1 | CK5_36590 | Pyruvate metabolism,Propanoate metabolism,Butanoate metabolism,Metabolic pathways,Microbial metabolism in diverse environments |
| *Ruminococcus_obeum* | WP_005422114.1 | CK5_34170 | ABC transporters |
| *Ruminococcus_obeum* | WP_005423288.1 | CK5_14710 | Purine metabolism,Pyrimidine metabolism,Metabolic pathways,RNA polymerase |
| *Ruminococcus_obeum* | WP_005423870.1 | CK5_11000 | Two-component system |
| *Ruminococcus_obeum* | WP_005424267.1 | CK5_32710 | Pentose and glucuronate interconversions,Metabolic pathways |
| *Ruminococcus_obeum* | WP_005424767.1 | CK5_21760 | RNA degradation |
| *Ruminococcus_obeum* | WP_044925681.1 | CK5_20370 | Aminoacyl-tRNA biosynthesis |
| *Ruminococcus_obeum* | WP_005424973.1 | CK5_20580 | Ribosome |
| *Ruminococcus_obeum* | WP_005424974.1 | CK5_20590 | Ribosome |
| *Ruminococcus_obeum* | WP_005425364.1 | CK5_28620 | One carbon pool by folate,Metabolic pathways,Microbial metabolism in diverse environments,Carbon metabolism |
| *Ruminococcus_obeum* | WP_044925744.1 | CGC63_07970 | Microbial metabolism in diverse environments,Carbon metabolism |
| *Ruminococcus_obeum* | WP_005425650.1 | CK5_01170 | Selenocompound metabolism,Aminoacyl-tRNA biosynthesis |
| *Ruminococcus_obeum* | WP_005425942.1 | CK1_08860 | Galactose metabolism,Amino sugar and nucleotide sugar metabolism,Metabolic pathways |
| *Ruminococcus_obeum* | WP_044925873.1 | CGC63_14915 | Aminoacyl-tRNA biosynthesis |
| *Ruminococcus_obeum* | WP_044925952.1 | CK5_07310 | Glycolysis / Gluconeogenesis,Citrate cycle (TCA cycle),Pyruvate metabolism,Metabolic pathways,Biosynthesis of secondary metabolites,Biosynthesis of antibiotics |
| *Ruminococcus_obeum* | WP_005427644.1 | CK5_18690 | ABC transporters |
| *Ruminococcus_obeum* | WP_005427646.1 | CK5_18680 | ABC transporters |
| *Ruminococcus_obeum* | WP_022387934.1 | A4V08_30895 | ABC transporters |
| *Ruminococcus_obeum* | WP_005427731.1 | CK5_18230 | Fructose and mannose metabolism,Microbial metabolism in diverse environments |
| *Ruminococcus_obeum* | WP_005427881.1 | CK5_17670 | Ribosome |
| *Ruminococcus_obeum* | WP_005428668.1 | CK5_21150 | Aminoacyl-tRNA biosynthesis |
| *Ruminococcus_obeum* | WP_005421511.1 | CK5_35020 | Purine metabolism,Pyrimidine metabolism,Mismatch repair,DNA replication |
| *Ruminococcus_obeum* | WP_005423866.1 | CK5_10990 | Ribosome |
| *Ruminococcus_obeum* | WP_005424878.1 | RTO_10100 | Quorum sensing |
| *Ruminococcus_obeum* | WP_005425256.1 | CK5_00890 | Ribosome |
| *Ruminococcus_obeum* | WP_005425370.1 | CK5_28650 | Microbial metabolism in diverse environments,Carbon metabolism |
| *Ruminococcus_obeum* | WP_005425377.1 | CK5_28700 | Methane metabolism,Microbial metabolism in diverse environments,Carbon metabolism |
| *Ruminococcus_obeum* | WP_022389420.1 | CK5_24860 | Taurine and hypotaurine metabolism,Pyruvate metabolism,Methane metabolism,Propanoate metabolism,Carbon metabolism |
| *Ruminococcus_obeum* | WP_044925805.1 | EUBELI_01806 | Purine metabolism,Metabolic pathways |
| *Ruminococcus_obeum* | WP_005426079.1 | CC1_26510 | Pyruvate metabolism |
| *Ruminococcus_obeum* | WP_005426368.1 | CK5_31560 | Arginine biosynthesis,Nitrogen metabolism,Biosynthesis of amino acids |
| *Ruminococcus_obeum* | WP_005427643.1 | CK5_18700 | ABC transporters,Bacterial chemotaxis |
| *Ruminococcus_obeum* | WP_005428565.1 | CK5_20850 | Ribosome |
| *Ruminococcus_obeum* | WP_005428696.1 | CK5_21480 | Purine metabolism,Alanine, aspartate and glutamate metabolism,Biosynthesis of antibiotics |
| *Eubacterium_siraeum* | WP_005354788.1 | ES1_19200 | Glycolysis / Gluconeogenesis,Biosynthesis of antibiotics,Carbon metabolism,Biosynthesis of amino acids |

**Supplementary Figure 1. Phylogenic trees based on whole genome SNP sites of remaining 6 species (mutated allele frequency exceeds 0.5).**


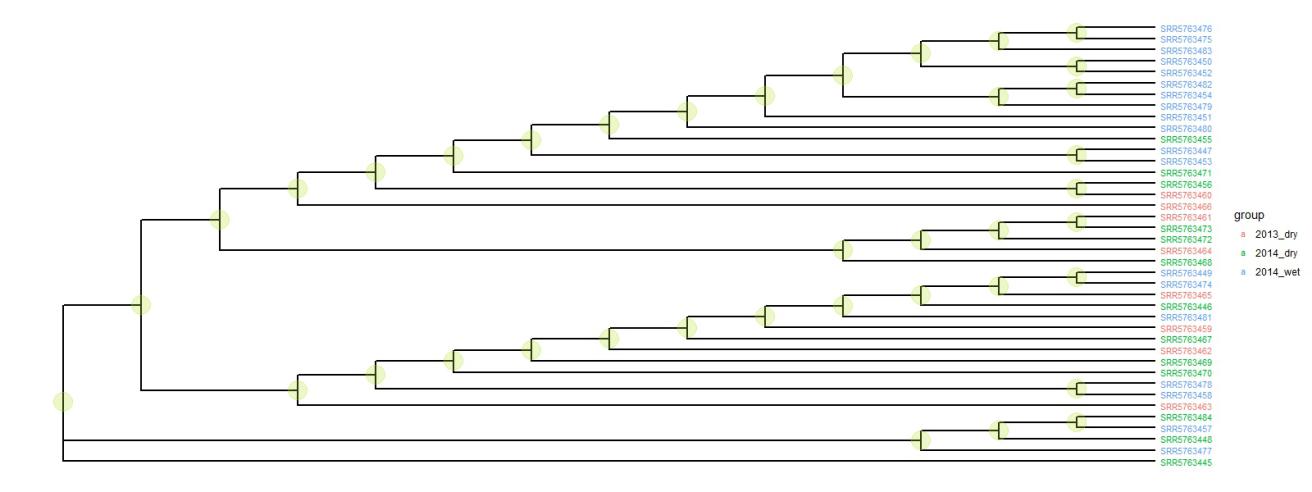


Supplementary Figure 1-1 Phylogenic tree of *Ruminococcus bromii*


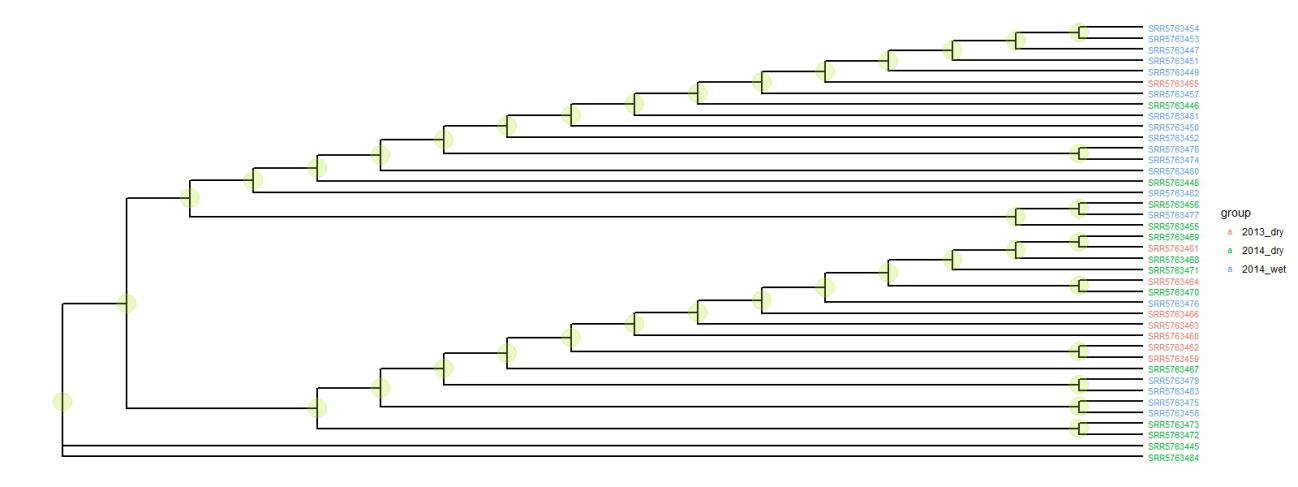


Supplementary Figure 1-2 Phylogenic tree of *Ruminococcus obeum*


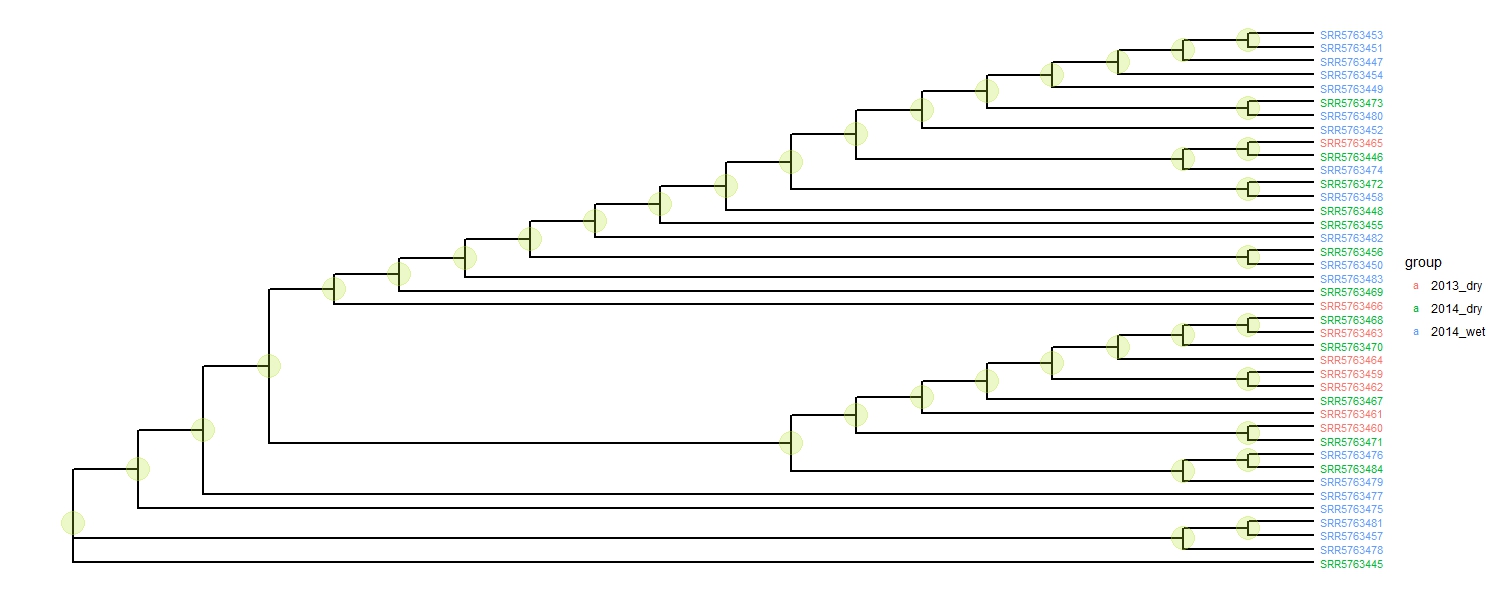


Supplementary Figure 1-3 Phylogenic tree of *Anaerostipes hadrus*


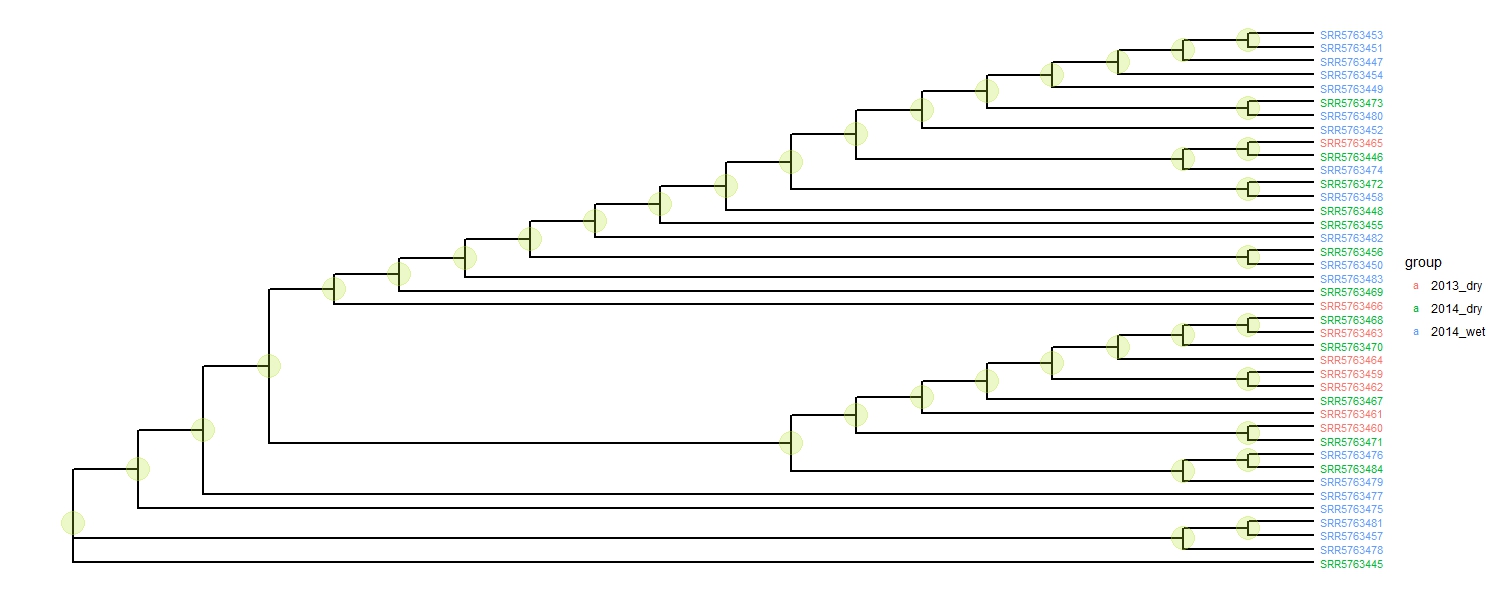


Supplementary Figure 1-4 Phylogenic tree of *Coprococcus comes*


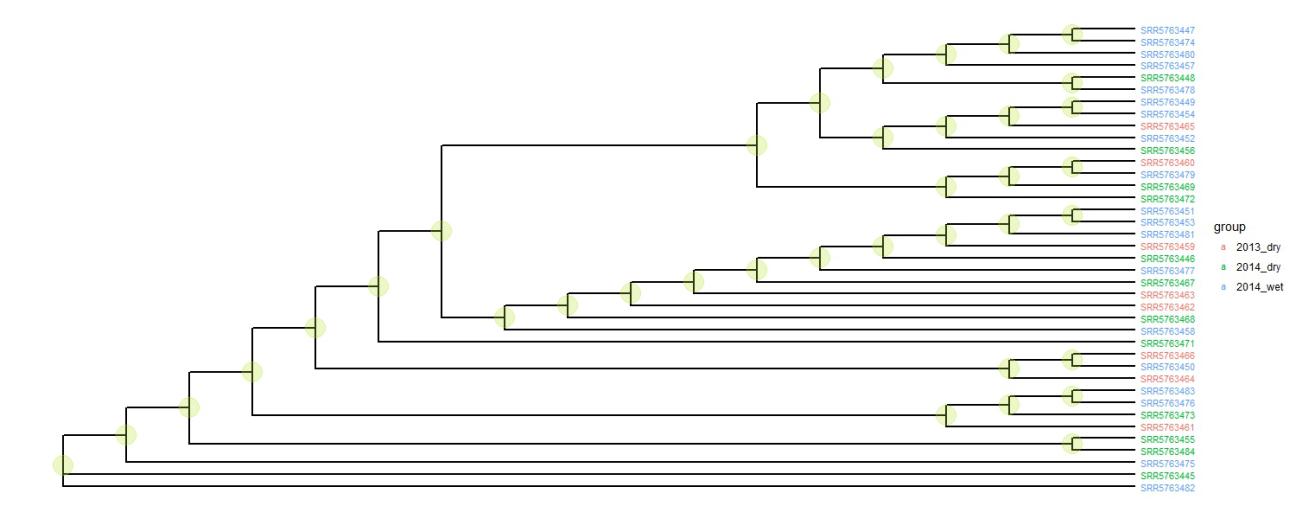


Supplementary Figure 1-5 Phylogenic tree of *Catenibacterium mitsuokai*


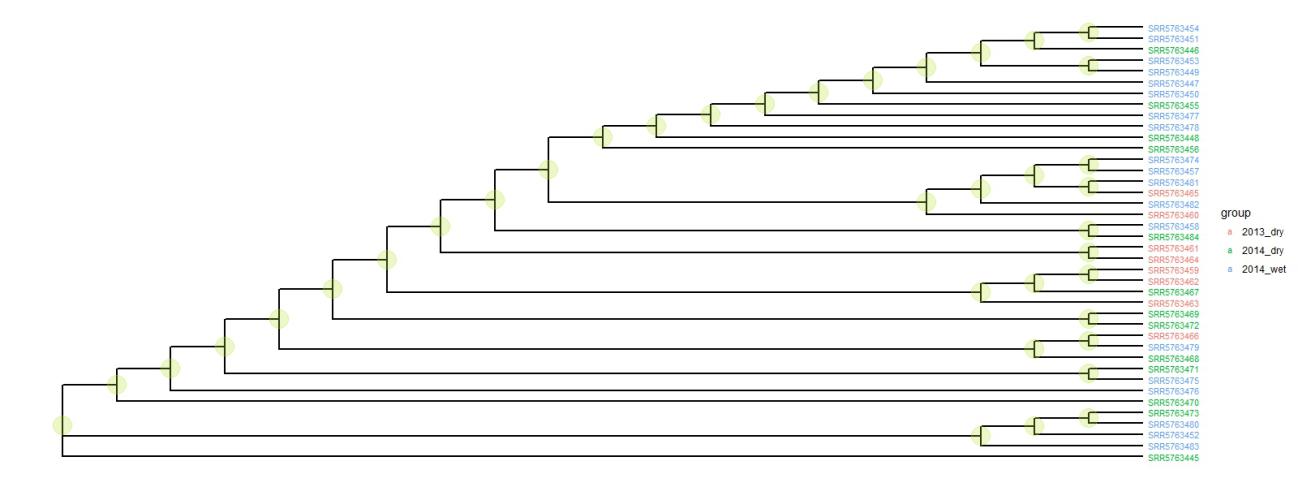


Supplementary Figure 1-6 Phylogenic tree of *Roseburia inulinivorans*

**Supplementary Figure 2. Clustering results of remaining six species based on whole genome SNP sites (mutated allele frequency bigger than 0.2).**


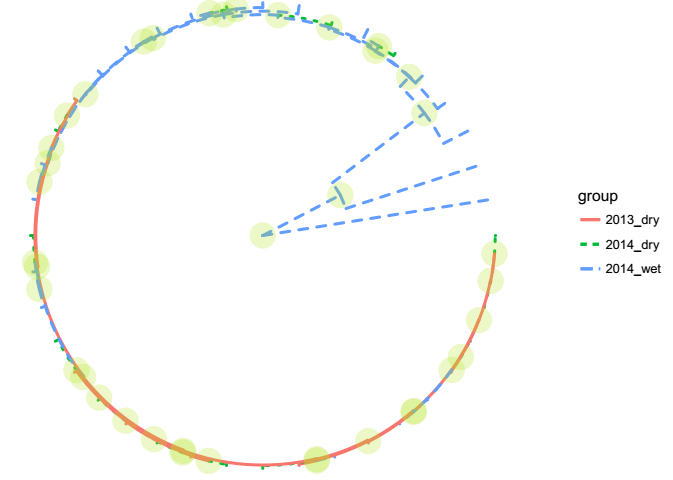


Supplementary Figure 2-1 Clustering result of *Ruminococcus bromii*


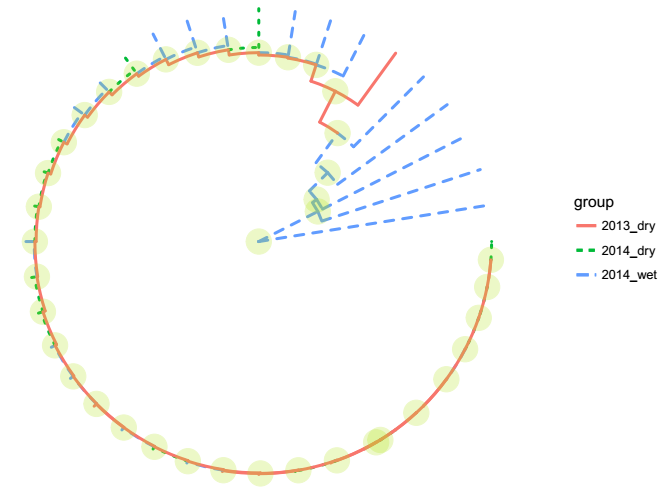


Supplementary Figure 2-2 Clustering result of *Ruminococcus obeum*


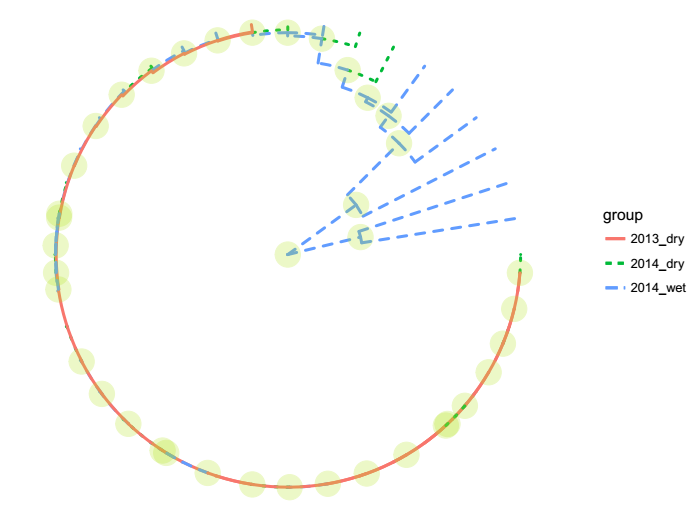


Supplementary Figure 2-3 Clustering result of *Anaerostipes hadrus*


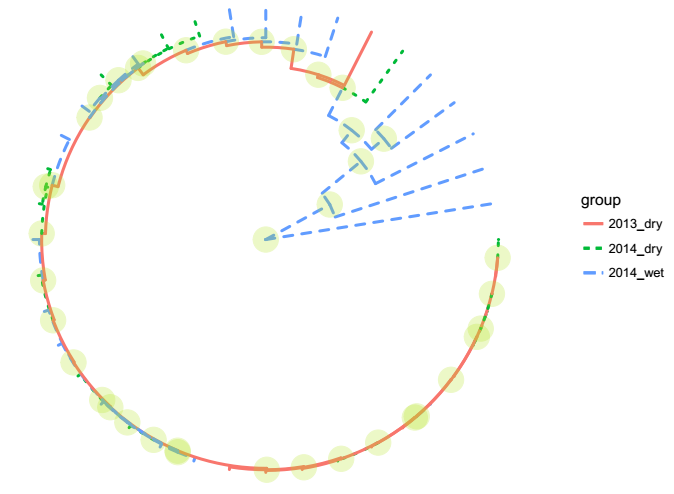


Supplementary Figure 2-4 Clustering result of *Coprococcus comes*


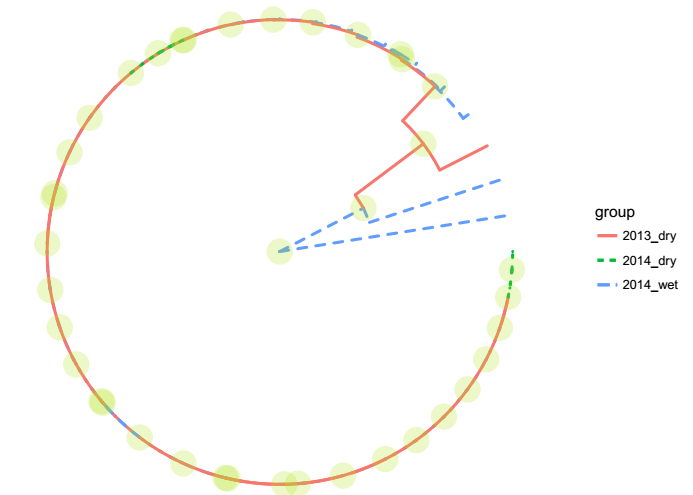


Supplementary Figure 2-5 Clustering result of *Catenibacterium mitsuokai*


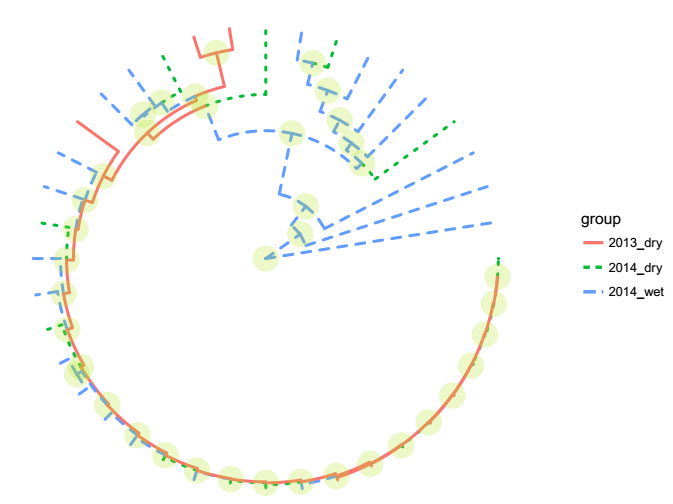


Supplementary Figure 2-6 Clustering result of *Roseburia inulinivorans*
